# Posttraumatic Orbital Emphysema: A Numerical Model

**DOI:** 10.1155/2014/231436

**Published:** 2014-09-16

**Authors:** Andrzej Skorek, Paweł Kłosowski, Łukasz Plichta, Dorota Raczyńska, Marcin Zmuda Trzebiatowski, Paweł Lemski

**Affiliations:** ^1^Otolaryngology Department, Medical University of Gdansk, Smoluchowskiego Street 17, 80-214 Gdańsk, Poland; ^2^Faculty of Civil and Environmental Engineering, Gdansk University of Technology, Poland; ^3^Ophthalmology Department, Medical University of Gdansk, Poland

## Abstract

Orbital emphysema is a common symptom accompanying orbital fracture. The pathomechanism is still not recognized and the usually assumed cause, elevated pressure in the upper airways connected with sneezing or coughing, does not always contribute to the occurrence of this type of fracture. Observations based on the finite model (simulating blowout type fracture) of the deformations of the inferior orbital wall after a strike in its lower rim. Authors created a computer numeric model of the orbit with specified features—thickness and resilience modulus. During simulation an evenly spread 14400 N force was applied to the nodular points in the inferior rim (the maximal value not causing cracking of the outer rim, but only ruptures in the inferior wall). The observation was made from 1 · 10^−3^ to 1 · 10^−2^ second after a strike. Right after a strike dislocations of the inferior orbital wall toward the maxillary sinus were observed. Afterwards a retrograde wave of the dislocation of the inferior wall toward the orbit was noticed. Overall dislocation amplitude reached about 6 mm. Based on a numeric model of the orbit submitted to a strike in the inferior wall an existence of a retrograde shock wave causing orbital emphysema has been found.

## 1. Introduction

Although orbital emphysema is a relatively common symptom following orbital trauma its pathophysiology still remains vague. Two main mechanisms explaining the transition of the air into the orbit have been described. The first mechanism is secondary to the rise of the air pressure in the upper airways during intensive nose blowing, sneezing, or coughing which leads to pumping the air into the orbit. The second mechanism refers to the theory of the so-called “shock wave.” Other causes such as iatrogenic, infectious, barotraumas, deliberate self-harm, and esophagus rupture are of rare occurrence.

## 2. Aim of the Study

In order to describe the behavior of the orbital walls after a trauma, a finite numerical method has been applied.

## 3. Material and Methods

A finite element model of the left orbital walls consisting of 969 triangle elements has been constructed in AutoCAD system. The shape of the model is based on CT scan patients who appeared at the Clinical Emergency Department of the Medical University of Gdańsk for neurological reasons (headaches, dizziness, status after an epileptic seizure, and suspected circulatory disorders within the CNS). The group included 100 patients (50 women and 50 men) aged between 18 and 93 years (60.3 years in average). All patients agreed to participate in the study. The CT scans were performed in three planes, horizontal, sagittal, and frontal, in 0.6 mm layers. The final version of the model included the average size and thickness of the orbital wall. The model was imported to MSC.Marc/Mentat system (thin shell elements). The fixed displacements boundary conditions have been applied in all nodes where orbital walls were linked to the remaining parts of the skull.

The measurements of Young's modulus were performed in a universal testing machine with an extensometer, on bone fragments sampled from the superior and medial walls of the orbit from cadavers who died from unnatural causes. The mean result obtained in the examined samples was 1.20 · 10^9^ N/m^2^.

In order to create a reliable model of the behavior of the inferior orbital wall after a trauma, we assessed its displacement in a numerical method (a dynamic variant of calculation) after applying a total force of 14400 Newton (N) (a thrust in 6 points with equal force of 2400 N). On the basis of other authors' [1–4] experience we assumed that the maximal moment of the force began after 1.3 · 10^−3^ s from the thrust and the force stopped acting on the bone model after 2.6 · 10^−3^ s. After applying a force stronger than 14000 N, destruction encompassed also the orbital rim [1, 2, 5, 6]. Thereafter a displacement of the inferior orbital wall upwards and downwards was observed in subsequent time periods. The analysis of the displacement was conducted in relation to the sagittal axis of the orbit beginning with latency *t* = 1 · 10^−3^ s to *t* = 1 · 10^−2^ s (with interval every Δ*t* = 1 · 10^−3^ s). The figures show graphical representation of the process.

## 4. Results

**Figure 1:**
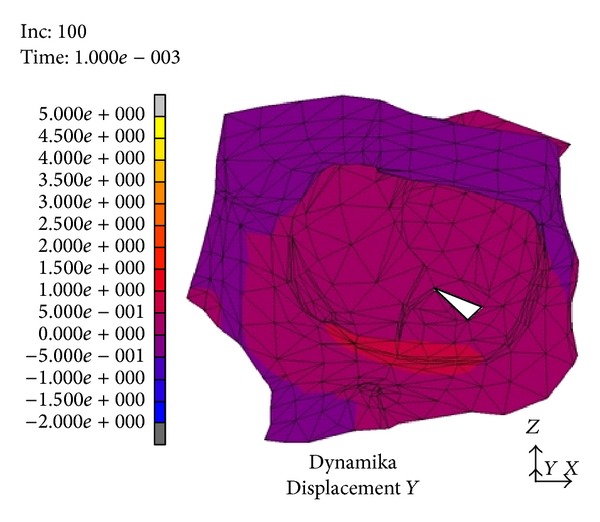
Dislocation after *t* = 1 · 10^−3^ s from the strike.

**Figure 2:**
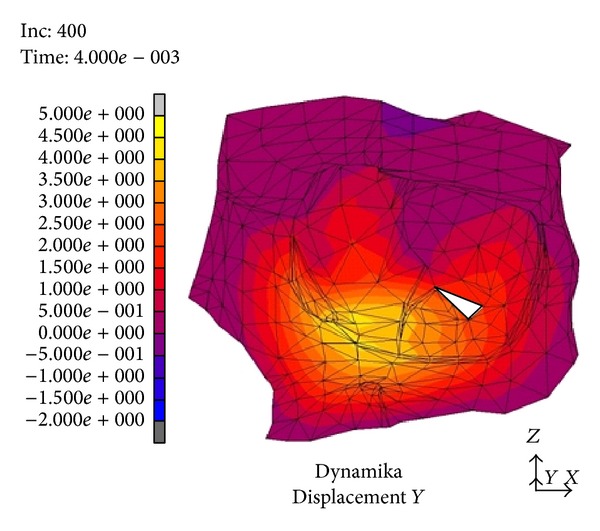
Dislocation after *t* = 4 · 10^−3^ s from the strike.

After the latency of *t* = 1 · 10^−3^ s from the thrust we observed a 1 mm displacement of the floor of the orbit downwards to the maxillary sinus (Figure 1). It included the whole bony rim of the orbit. In the following time periods the distances reached 4–4.5 mm and gradually encompassed the inferior wall, the base of the medial and lateral wall, and finally the frontal wall of the maxillary sinus. The maximal values of the displacement were measured after *t* = 4 · 10^−3^ s from the beginning of the thrust (Figure 2). After the time of *t* = 5 · 10^−3^ s maximal displacements were observed (4-5 mm).

**Figure 3:**
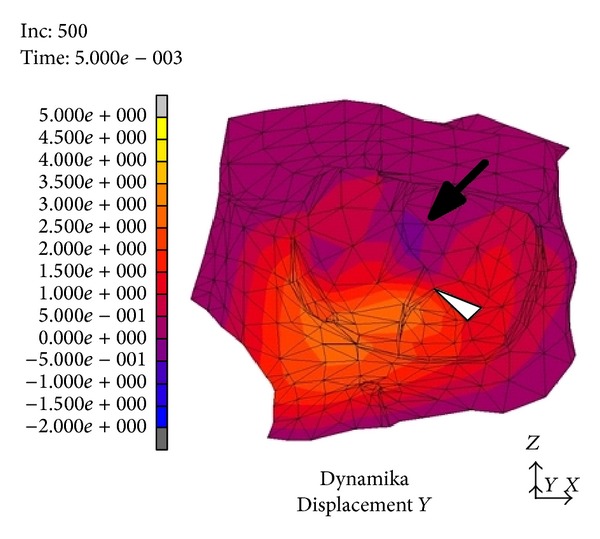
Dislocation after *t* = 5 · 10^−3^ s from the strike. Black arrow indicates the dislocation directed towards the inside of the orbit, the beginning of the “retrograde wave.”

**Figure 4:**
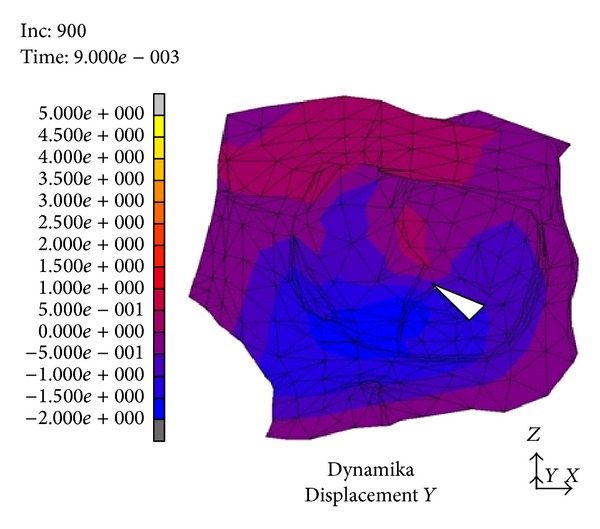
Dislocation after *t* = 9 · 10^−3^ s from the strike.

**Figure 5:**
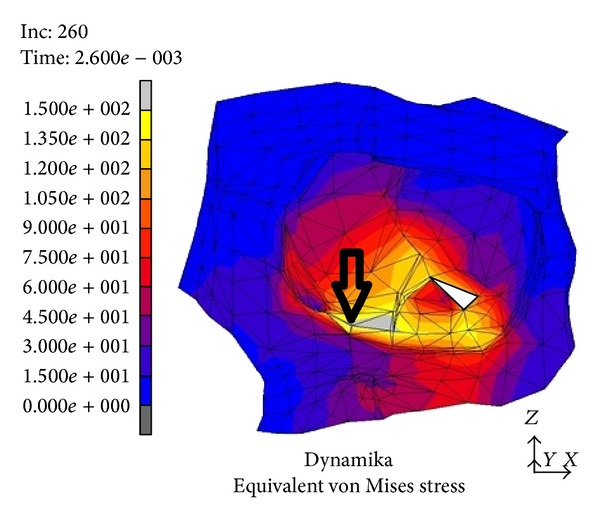
Location of point A in the inferior wall, which served for assessment of the dislocation.

**Figure 6:**
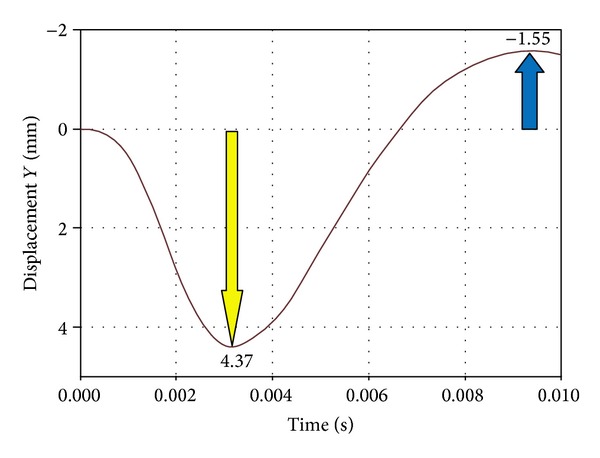
Dislocation evaluated in point A in relation to the time after a strike.

In the area medial to the suborbital nerve canal and in the medial part of the orbit occurred a deformity sized 2.5 to 3 mm. It was directed outwards, whereas in the posterior part of the orbital floor (between the inferior orbital fissure and the optic nerve canal) the area of relocation extended as far as 0.5 mm and was directed towards the inside of the orbit, “the recurring wave” (white arrow, Figure 3). In the subsequent time periods we noticed the accretion of the displacement directed towards the inner orbit occurring in the suborbital nerve canal area; then, it involved the whole inferior wall and bases of the medial and lateral walls (the picture analogical to the displacements directed outwards) (Figure 4). Maximal displacements were observed at *t* = 8 · 10^−3^ s and *t* = 9 · 10^−3^ s and valued 2 mm. At the time of *t* = 2.6 · 10^−3^ s the stress in the inferior orbital wall exceeded the assumed limit value which clinically entails orbital wall fracture (Figure 5). Displacements in point A (localized in frontal part of the floor) are presented in Figure 6.

## 5. Discussion

Pathological presence of the air in soft tissues of the orbit (*orbital emphysema, pneumoorbita*), eyelids, or the face is commonly caused by different types of bone fractures including blowout fracture [7–10]. According to Key et al. [11] up to 50% of the cases of blowout fracture are accompanied by orbital emphysema (the air may collect in the preseptal or retroseptal regions of the orbit or subcutaneously). The air in this type of trauma enters the orbit from the ethmoid, maxillary, and frontal sinuses. Although the pathomechanism of the orbital emphysema is well documented in case of barotrauma in divers and after surgical orbital decompression (e.g., in Graves-Basedow disease) [12, 13], in case of blowout its occurrence still remains unclear. The normal pressure in the confined orbital space ranges from 15 to 20 mm Hg. In order for the air to escape from the sinuses to other regions a force there must appear either pushing it out or sucking in into surrounding tissues. The air can be compressed in the sinuses on condition that their natural openings are obstructed, for example, in case of infection or trauma, and it exits the sinus through a natural defect in its wall or a posttraumatic fissure [7, 14, 15]. In case of normal sinus openings the air pressure in the sinuses may elevate during vomiting, coughing, or sneezing or when an individual is subjected to a high external pressure, for example, in case of diving or travelling by plane [7]. The air may relocate right after a trauma or within two weeks, which is connected with a formation of scarce obturating the sinuses [8]. Heerfordt in a study performed on cadavers (according to [12]) established the maximal pressure not causing any rupture of the orbital periosteum in younger and older people, which is 70 to 100 mm Hg and 10 to 15 mm Hg, respectively. The results of this research carry two implications. In older people the air may more easily get to the orbit in comparison to younger people, whereas in younger individuals with orbital emphysema it comes more frequently to loss of vision which is connected with higher pressure rupturing the periosteum and in consequence causing occlusion in the central retinal artery.

Can the air be sucked into the orbit? The pressure rupturing the bony walls of the orbit in hydraulic model propagates from the inside to the outside. The eyeball dislocating posteriorly compresses the orbital soft tissues which exit the orbit through the bony fracture creating a hernia. In such an explanation of the phenomenon there is no place for occurrence of a retrograde pressure. However, in the experimental research on cadavers Walzer (according to [16]) observed orbital emphysema in 6 out of 7 cases after a punch in the eyeball (no elevation of pressure in the nasosinal space was possible). There are numerous examples of presence of air in the orbit and/or cranial vault confirmed in posttraumatic unconscious patients or in those not clearing the nose directly after the trauma.

In a numeric model (dynamic variant) after a strike in the inferior orbital rim it comes to rise of mechanical stress and occurrence of dislocations which create a “shock wave” (Figures 1–4). The dislocation area and its size were increasing with time and progressed towards the inside of the orbit encompassing middle and lateral walls. In the first period from the impact the stress was directed to the maxillary sinus. The maximal amount of dislocation and its area was reached after *t* = 4 · 10^−3^ s (Figure 2) and involved the whole area of the front part of the orbital floor (from the orbital ostium of the nasolacrimal canal via the region of the suborbital nerve to the joint of the inferior with lateral orbital wall). An interesting finding is occurrence of the “retrograde wave,” that is, the area of the dislocation directed toward the inside of the orbit. This area appears after *t* = 5 · 10^−3^ s (Figure 3) in the region between optic canal and inferior orbital fissure and it spreads initially to the medial wall (inferoposterior part) and subsequently to the orbital floor. Maximal dislocation reaches 2 mm and is directed towards the inside of the orbital. The maximal dislocation area is observed after *t* = 9 · 10^−3^ s (Figure 4). Interesting parallels are found between images after *t* = 3 · 10^−3^ s and *t* = 8 · 10^−3^ s, as well as after *t* = 4 · 10^−3^ s and *t* = 9 · 10^−3^ s. Maximal dislocations directed towards the outside of the orbit (4–4.5 mm) and the inside (2 mm) are shown in Figures 2 and 4. In both figures the amount of dislocation is similar but they are directed oppositely. In Figure 6 the dislocation in the frontomedial area of the orbit (point A) is presented. The difference between the dislocation directed to the outside (4.37 mm) and to the inside of the orbit (1.55 mm) values 5.92 mm. The dislocation of the bony wall towards the maxillary sinus is accompanied by the elevation of the pressure inside of the orbit and its drop on the other side and reversely. The dislocation of the bony wall towards the maxillary sinus is accompanied by increased pressure in the orbit and decreased on the other side of the wall. The retrograde shock wave causes pushing of the wall towards the inside of the orbit and reversal of the pressure gradient and in consequence sucking the air into the orbit. If in addition a fracture occurs after *t* = 2.6 · 10^−3^ s in the inferior wall it can be assumed that elevated periosteum of the orbit (sometimes already ruptured) may create a space (extra- or subperiosteal) in which the air from the sinuses is squeezed. This hypothesis was introduced by Fuchs in 1901 (according to [16]) who suspected that the eyeball shifting towards the inside of the orbit hits the delicate medial wall causing its fracture and aspiration of the air into the orbit. Progressing along the bony wall energy causes occurrence of spaces with increased (above the bony wall) and decreased (under the bony wall) pressure. These spaces are observed in the orbit and in nasal sinuses, alternately. When a region with decreased pressure abuts a fractured bone separating the orbit from the sinuses, it may come to relocation of the air to the orbit (drawing in), which is instantaneously bested by surrounding soft tissues. Due to the use of numeric model it was possible to confirm such mechanism of occurrence of orbital emphysema. The air usually locates close to the sinus from which it escaped. The patient with orbital emphysema may present with exophthalmos or double or deterioration of vision. The valve mechanism leads to the increase of pressure in the orbital tissues above 65–70 mm Hg, which prevents blood from flowing through the middle retinal artery (in the research using animals this pressure reaches 105 mm Hg [17, 18]). Burt et al. [7] described posttraumatic deterioration of the eyeball movement due to the impression of the collected air spaces in the orbit. The treatment in case of orbital emphysema accompanied by increasing symptoms (especially deterioration of vision) should include one of the procedures: lateral canthotomy, inferior and/or superior cantholysis, orbital decompression, or simple puncture of the air vessel which may prevent the patient from blindness [11, 12, 15, 19]. When no serious symptoms are involved the treatment should be limited to antibiotic therapy, antiemetic drugs, pain killers, and hospitalization. Steroids should be administered in case of a hematoma and/or occurrence of air space in retrobulbar region [20]. The study we have performed allowed us to describe in a numeric model a sequence of events after a strike in the inferior wall of the orbit. Nagasao et al. [2, 5] suspected that after a punch in the orbital floor its frontal part is dislocated towards the maxillary sinus, whereas the posterior one on the contrary is dislocated towards the inside. These observations were based on the analysis of the morphological structure of the orbital floor and studies of other authors [21–23]. More frequent occurrence of fractures in the frontal part of the orbital floor is explained by the difference in bony thickness which is greater in the posterior part. Our research (analysis of the dynamic numeric model) does not confirm such a behavior of the inferior orbital wall, whereas it proves occurrence of a retrograde wave causing deformation of the inferior wall. Also our attention is directed to a relatively large amplitude of tilting (6 mm) (Figure 6). However in the presented study we did not include the moment of fracture after *t* = 2.6 · 10^−3^ s which in consequence leads to larger dislocation in subsequent time periods.

## 6. Summary 

The air trapped inside of the orbit is a complication after a blowout fracture. The reason for its relocation from the surrounding sinuses is the elevation of pressure due to coughing or sneezing. Based on a numeric model of the orbit submitted to a strike in the inferior wall, an existence of a retrograde shock wave which may be responsible for occurrence of orbital emphysema has been found.
